# Molecular phylogenetics of *Micromeles* (Rosaceae: Maleae): implications for taxonomy

**DOI:** 10.1186/s12870-025-07143-z

**Published:** 2025-08-27

**Authors:** Junling Hu, Baomei Tan, Xin Chen, Mengdie Dong, Jianhui Ma

**Affiliations:** Co-Innovation Center for Sustainable Forestry in Southern China, College of Life Sciences, Nanjing Forestry University, Nanjing, China

**Keywords:** *Micromeles*, Plastid genomes, ITS sequences, Phylogeny, Taxonomy

## Abstract

**Background:**

*Micromeles* Decne., belonging to the tribe Maleae of the family Rosaceae, has a complicated taxonomic history. It was either recognized as a distinct genus in the broad sense (*Micromeles s.l.*), or in the restricted sense (*Micromeles s.s.*), or even merged within the genus *Aria*, *Pyrus*, or *Sorbus*. The present study aims to clear the confusions of the taxonomic identity of *Micromeles* using both plastid and nuclear datasets.

**Results:**

Both phylogenetic trees based on plastome and ITS datasets strongly support the monophyly of *Micromeles s.l.* However, *Micromeles s.s.* and the other five small genera, including *Alniaria*, *Dunnaria*, *Griffitharia*, *Thomsonaria*, and *Wilsonaria* are non-monophyletic and are synonymized under *Micromeles s.l. Pleiosorbus* has been synonymized under *Micromeles s.l.* following Lu and Ku, and Mezhenska et al. based on their morphological similarities. Two new combinations at the infraspecific level are proposed here.

**Conclusions:**

This study approves of the adoption of the broad sense of *Micromeles* based on both phylogenetic and morphological evidence.

**Supplementary Information:**

The online version contains supplementary material available at 10.1186/s12870-025-07143-z.

## Introduction

The genus *Micromeles* Decne. was established by Decaisne [1], who stated that it was closely related to *Aria* (Pers.) J.Jacq. ex Host, but differed in having much smaller flowers and pomes, inferior ovaries with epigynous discs, and deciduous calyxes after flowering. However, the taxonomic status of *Micromeles* has been controversial for a long time since its establishment. It was either recognized as a distinct genus in the restricted sense (*Micromeles s.s.*) including only the simple-leaved species with deciduous calyxes [1, 2], or in the broad sense (*Micromeles s.l.*) comprising all the Asiatic simple-leaved species with both deciduous and persistent calyx [3], or even merged within the genus *Aria*, *Pyrus*, or *Sorbus*.

Along with *Aria* and *Sorbus s.s.* (which includes only the pinnate-leaved species), Hooker [4] reduced *Micromeles* to *Pyrus* as sections, and his treatment was followed by Christenhusz et al. [5]. Wenzig [6] united *Micromeles* and *Aria* with *Sorbus s.l.* (which includes both the pinnate-leaved species and the simple-leaved species), and his treatment was followed by Rehder [7], Yu and Kuan [8], Yu and Lu [9], Gabrielian [10], and Aldasoro et al. [11]. Koehne [12, 13] maintained both *Micromeles* and *Aria* as distinct genera and treated as *Sorbus* in the restricted sense in his revision of the entire Maloideae (i.e., Maleae). Koehne [12, 13] diagnosed “deciduous calyx” as the key morphological character of *Micromeles*, and transferred three *Aria* species with deciduous calyx, including *A. alnifolia* (Sieb. & Zucc.) Rushforth, *A. japonica* Decne, and *A. tiliifolia* Decne to *Micromeles*, and his concept was followed by Kovanda and Challice [2]. Hedlund [14] also kept *Micromeles* as a separate genus but merged *Aria* into *Sorbus s.l.*, and his treatment was followed by Schneider [15] and Kalkman [16, 17]. Phipps et al. [18] reduced *Micromeles* as a subgenus of *Sorbus*, namely subg. *Micromeles* (Decne.) Phipps, Robertson & Spongberg. Phipps et al. [18] and Robertson et al. [19] separated *Aria*, *Chamaemespilus* Medik., *Cormus* Spach, and *Torminalis* Medik. from *Sorbus* as four independent genera, but remained *Micromeles* within *Aria.* Phipps’s opinion was followed by Ohashi and Iketani [20], who later transferred all species previously placed in *Micromeles* to *Aria*, resulting in 36 new combinations.

Mezhenska et al. [3] expanded *Micromeles* (hereafter *Micromeles s.l.*) to include all the Asiatic simple-leaved species previously placed in *Sorbus s.l*. In contrast, Rushforth [21] restricted *Micromeles* to accommodate six species (hereafter *Micromeles s.s.*), *M. cuspidata* (Bertol.) C.K.Schneid., *M. griffithii* Decne, *M. paucinerva* (Merr.) Mezhenskyj, *M. polycarpa* (Hook.f.) Panigrahi, *M. rhamnoides* Decne., and *M. salwinensis* (T.T. Yu & L.T.Lu) Mezhenskyj. He [21] re-defined the genus based on the characteristics of small, 4–7 mm fruits without lenticels, two co-lateral ovules in the two carpels, craspedodromous venation (secondary veins reach the leaf edge), deciduous or persistent calyx. He [21] resurrected the genus *Pleiosorbus* L.H.Zhou & C.Y.Wu and described five new small genera, *Alniaria* Rushforth, *Dunnaria* Rushforth, *Griffitharia* Rushforth, *Thomsonaria* Rushforth, and *Wilsonaria* Rushforth. He assigned the remaining Asiatic simple-leaved species in *Sorbus s.l.* to these six genera. Consequently, *Micromeles s.l.* defined by Mezhenska et al. [3] was separated into seven small genera by Rushforth [21].

Molecular phylogenetic approaches have been extensively utilized to elucidate evolutionary relationships among diverse plant taxa [22]. However, previous molecular studies mainly focused on the phylogeny of the tribe Maleae, with few concentrated specifically on the circumscription and infrageneric relationships within *Micromeles s.l.* No taxonomic revision was proposed for the limited samples employed, and for the conflicting topologies between cpDNA and the internal transcribed spacer (ITS) data [23, 24]. Studies based on ITS sequences, with only one *Micromeles* species (*A. alnifolia*) by Campbell et al. [25] and Campbell et al. [26] resolved it as a sister to *Heteromeles* M.Roem.; with six *Micromeles s.l.* species by Li et al. [27] resolved it as monophyletic and sister to *Aronia* Mitch. A combination of 11 chloroplast regions plus ITS sequences of 15 *Micromeles s.l.* species by Lo and Donoghue [23] resolved it as monophyletic but sister to *Sorbus s.s.* and *Aria* in chloroplast and ITS phylogeny, respectively. The phylogenetic study of *Sorbus s.l.* based on ITS sequences with 22 *Micromeles s.l.* species in the genus name of *Sorbus* resolved it as monophyletic and sister to *Eriobotrya* Lindl. [28]. Our previous phylogenomic study based on the whole plastomes strongly supported that *Micromeles s.l.* was monophyletic and was the sister group of *Sorbus s.s.* [24].

In this study, we obtained plastome and ITS datasets of an expanded sampling of *Micromeles s.l*. and of the reportedly genera within the tribe Maleae. The aims are to: (1) test the monophyly of *Micromeles s.s.* and its allies, *Alniaria*, *Dunnaria*, *Griffitharia*, *Thomsonaria*, and *Wilsonaria*; (2) delineate taxonomic boundaries of *Micromeles*; (3) investigate the phylogenetic relationships of *Micromeles s.l.* within the tribe Maleae.

## Methods

### Taxon sampling

For plastid-based phylogenetic inference, we used a total of 105 samples: 60 ingroup samples (representing 30 species and one unidentified species from six small genera) and 45 outgroup samples representing 41 species from 35 genera. A total of 14 plastomes were newly sequenced in this study, and 91 samples were obtained from GenBank. For ITS-based phylogenetic inference, we used a total of 106 samples, comprising 60 ingroup samples representing 27 species and one unidentified species from six small genera, and 46 outgroup samples representing 42 species from 35 genera. A total of 30 ITS sequences were newly recovered while the others were retrieved from GenBank (see Table S1). Outgroups include all other currently recognized genera (27 genera) within Maleae, the tribes Amygdaleae and Sorbarieae in Amygdaloideae, Roseae in Rosoideae, and *Ulmus macrocarpa* Hance (Ulmaceae). One to five accessions per species were examined. Leaf samples of *Micromeles s.l.* species were collected in the field between 2017 and 2023 from Anhui, Guizhou, Hubei, Shanxi, Sichuan, Xizang, Yunnan, and Zhejiang Provinces in China. Fresh leaves were immediately dried with silica gel for further DNA extraction. Voucher specimens were deposited in the Herbarium of Nanjing Forestry University (NF) and collection information is listed in Table S1.

### DNA extraction, library preparation, sequencing, and assembly

Total genomic DNA was isolated from 20 mg silica-gel dried leaf tissue using the Super Plant Genomic DNA Kit (Polysaccharides and Polyphenolics-rich; TIANGEN, Beijing). A modified CTAB method [29] was additionally employed for samples requiring enhanced impurity removal, particularly when processing tissues with high secondary metabolite content where the standard kit protocol yielded insufficient DNA quality. The extracted genomic DNA was quantified by Qubit Fluorometer (Invitrogen). Samples meeting concentration standards (≥ 1 μg) were used for library construction with the BGI Optimal DNA Library Prep Kit, which performed integrated enzymatic fragmentation, end repair, and dA-tailing in a single step. Adapters were ligated using the BGI Plug-In Adapter Kit. Following manufacturer's protocol, libraries were size-selected for 250–350 bp inserts via two-round AMPure® XP bead purification (0.8 × followed by 0.2 × ratios). PCR amplification was performed for 6 cycles (98 °C for 10 s, 60 °C for 30 s, 72 °C for 30 s) using primers provided in the BGI Plug-In Adapter Kit. Final libraries were sequenced on the DNBSEQ platform (BGI) for 150-bp paired-end reads. Raw reads were quality-checked with FastQC v.0.11.9 [30] and processed with SOAPnuke v.1.5.6 [31] to remove adapter-contaminated reads, low-quality bases (Q < 20), and residual short reads (< 150 bp). Approximately 2–3 Gb clean data per sample were generated and the details were provided in Table S1.

The mapped plastid reads were assembled into contigs using the GetOrganelle v.1.7.5.3 [32] with the suitable parameter setting for *Micromeles* (cpDNA: wordsize = 103, round limit = 15, k-mer = 21, 45, 65, 85, 105, 125; ITS: round limit = 10, k-mer = 35, 85, 115.) by mapping the reads to reference genomes obtained from GenBank (cpDNA:*Torminalis glaberrima* (Gandoger 1875: 90) Sennikov & Kurtto (2017: 32), NC033975.1; ITS: *Malus ioensis* (Alph.Wood) Britton, MN215985.1) and the average base-coverages of the assembled genomes are provided in Table S1.

Reads were aligned to the assembled plastomes sequences using Bandage v0.8.1 [33] for visualization and processing to validate assembly accuracy. Subsequently, the relative positions and orientations of individual contigs were manually refined in Geneious v9.0.2 [34] by aligning them to the reference genome, ensuring proper genomic placement and structural consistency.

We annotated the plastid genome assemblies using both PGA v. 2019 [35] and GeSeq [36], with *Sorbus tianschanica* Rupr. (ON049666.1) as the reference. These annotations were subsequently manually checked and adjusted using Geneious v9.0.2. Additionally, we annotated the ITS sequences in Geneious v9.0.2 through reference-based alignment, utilizing the ITS sequence of *Malus ioensis* (Alph.Wood) Britton (MN215985.1) as the reference.

### Phylogenetic analyses

Phylogenetic analyses were performed using Bayesian inference (BI) and maximum likelihood (ML) methods for the plastomes and ITS sequence datasets. The two datasets were separately aligned using MAFFT v.7.037 [37] with manual adjustments when needed in Geneious v9.0.2. The best-fitting nucleotide substitution models for BI analyses were determined according to the Akaike Information Criteria (AIC) using ModelFinder v2.2.0 [38]. BI analysis of plastome and ITS was conducted in MrBayes v.3.2.7 [39] in GTR + F + I + G4 mode. The Markov chain Monte Carlo (MCMC) was carried out for 6,000,000 generations sampling trees every 1000th generation. The initial 25% of the sampled trees were discarded as burn-in, and posterior distributions were combined from both runs. The best-scoring Inferred ML trees for plastome and ITS based on 20 additive replicates using the GTR + GAMMA model in RAxML v.8.2.12 [40]. The topological robustness is evaluated by using rapid bootstrap algorithm with 1000 non-parametric bootstrap replicates. Both ML analyses and BI analyses were visualized by the FigTree v.1.4.3 (https://tree.bio.ed.ac.uk/software/Figtree/), with nodal support values indicated.

Support values represented by Bayesian posterior probability (PP) and Maximum likelihood bootstrap support (BS) of 0.90–0.94 | 75–89%, 0.95–0.99 | 90–99%, and 1.0 | 100%, are considered as moderate, strong, and highest, respectively [41, 42].

### Hypothesis tests

Alternative phylogenetic hypotheses were evaluated by conducting an approximately unbiased (AU) test [43]. Constraint trees were constructed in Mesquite v.3.8.1 [44] to reflect hypothesized relationships. A tree topology test was then performed using IQ-TREE v.3.0.1 (http://www.iqtree.org/doc/) to calculate the *p*-value for each alternative topology.

### Morphological data

To evaluate the morphology-based classification, five key characters were mapped onto trees based on plastome and ITS datasets, i.e., indumentum color, leaf venation type, calyx (deciduous or persistent), fruit color, and size. Qualitative characters such as indumentum color, leaf venation type, calyx deciduous or persistent, and fruit color were derived from observations during our field trips, based on description in the protologues when the species were published, and from images archived at the Plant Photo Bank of China (PPBC; http://ppbc.iplant.cn/) and the Global Biodiversity Information Facility (GBIF; https://www.gbif.org/). Fruit size was measured during our field trips, based on the description in protologues, flora'accounts and taxonomic literature [11, 45]. Information on the five morphological characters used in this study is provided in Table S2.

## Results

### Phylogenetic analyses

The size of the 14 newly sequenced plastomes ranges from 159,466 bp in both *Micromeles atrosanguinea* (T.T.Yu & H.T.Tsai) Mezhenskyj and *M. guanii* (Rushforth) Mezhenskyj to 160,517 bp in *M. alnifolia* var. *angulata* S.B.Liang (PQ899004.1). The recovered ITS sequences lengths are between 586 and 589 bp for all the samples.

### Plastome phylogenies

**Fig. 1:**
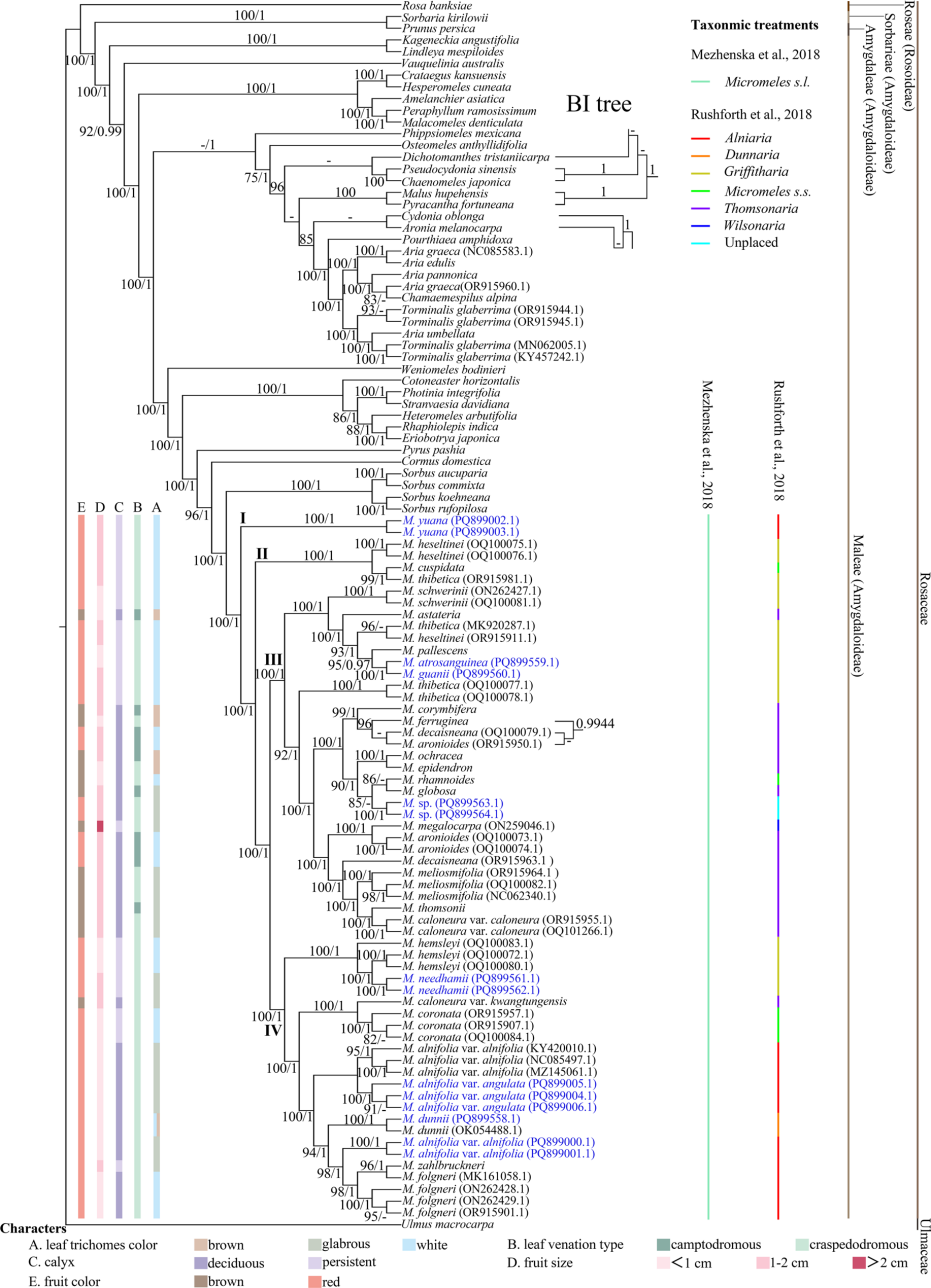
Phylogenetic tree inferred from the plastome dataset. Taxon names of newly generated sequences are indicated in blue. Support was only indicated for nodes that received support ≥ 75% BS for the maximum likelihood approach and ≥ 0.90 for the Bayesian analyses approach. Five morphological characters are mapped on the phylogenetic tree: **A** leaf trichomes color; **B** leaf venation type; **C** calyx deciduous or persistent; **D** fruit size; **E** fruit color

The plastome ML tree is generally congruent with the BI tree, with only minor discordances in the sister-group relationships among seven species of outgroup genera in Maleae and three species of ingroup (*M. aronioides* (Rehder) Kovanda & Challice, *M. decaisneana* (Lavallée) C.K.Schneid. (OQ100079.1) and *M. ferruginea* (Wenz.) Koehne; Fig. 1). Therefore, only the Bayesian topology is shown here with both BI/ML support values indicated at each node, along with the two different topologies for the ten species (Fig. 1).

*Micromeles s.l.* is monophyletic with highest support (100 | 1; Fig. 1). While the six small genera defined by Rushforth [21], *Alniaria*, *Dunnaria*, *Griffitharia*, *Micromeles s.s.*, *Thomsonaria*, and *Wilsonaria*, are recovered as non-monophyletic and intermingle together. *Micromeles s.l.* is sister to the pinnate-leaved *Sorbus s.s.*, and is well separated from the simple-leaved genera *Aria* and *Pyrus*. The other two simple-leaved genera formerly included in *Sorbus s.l.*, *Chamaemespilus* and *Torminalis*, are embedded within *Aria* with highest support (100 | 1).

In *Micromeles s.l.*, four highly-supported clades are revealed (I-IV; Fig. 1). *Micromeles s.s.* is represented by three species that are resolved to be isolated in three separate clades, i.e., the type species *M. rhamnoides* in clade III, *M. cuspidata* in clade II and *M. coronata* (Cardot) Mezhenskyj in clade IV. Two accessions of *M. yuana* (Spongberg) Mezhenskyj from *Alniaria* formed the clade I, while three other species from *Alniaria*, *M. alnifolia* (Siebold & Zucc.) Koehne, *M. folgneri* C.K.Schneid. and *M. zahlbruckneri* (C.K.Schneid.) Mezhenskyj, are resolved in clade IV. The monotypic genus *Dunnaria*, represented by two samples of *M. dunnii* (Rehder) Mezhenskyj, is nested within clade IV, alongside species from *Alniaria*, *Griffitharia*, *Micromeles s.s.*, and *Thomsonaria*. *Griffitharia* is represented by eight species that are resolved in three separate clades: two accessions of *M. heseltinei* (Rushforth) Mezhenskyj, one accession of *M. thibetica* (Cardot) Mezhenskyj nested in the clade II; three accessions of *M. hemsleyi* C.K.Schneid. and two accessions of *M. needhamii* (Rushforth) Mezhenskyj formed a group in clade IV; three accessions *M. thibetica* nested in clade III together with *M. atrosanguinea*, *M. guanii*, *M. pallescens* (Rehder) Mezhenskyj, and *M. schwerini* C.K.Schneid. *Thomsonaria* is represented here by 12 taxa, *M. aronioides*, *M. astateria* (Cardot) Mezhenskyj, *M. caloneura* var. *caloneura* Stapf, *M. caloneura* var. *kwangtungensis* T.T.Yu, *M. corymbifera* (Miq.) Kalkman, *M. decaisneana*, *M. epidendron* (Hand.-Mazz.) Kovanda & Challice, *M. ferruginea*, *M. globosa* (T.T.Yu & H.T.Tsai) Mezhenskyj, *M. meliosmifolia* (Rehder) Kovanda & Challice, *M. ochracea* (Hand.-Mazz.) Mezhenskyj, and *M. thomsonii* (King ex Hook.f.) C.K.Schneid. Although the 12 taxa are nested within clade III, with one *M. caloneura* var. *kwangtungensis* in clade IV. *Wilsonaria* is represented by the type species *M. megalocarpa* (L.H.Zhou & C.Y.Wu) Mezhenskyj that is deeply nested in clade III among species from *Griffitharia*, *Micromeles s.s.*, and *Thomsonaria*.

### ITS phylogenies

**Fig. 2:**
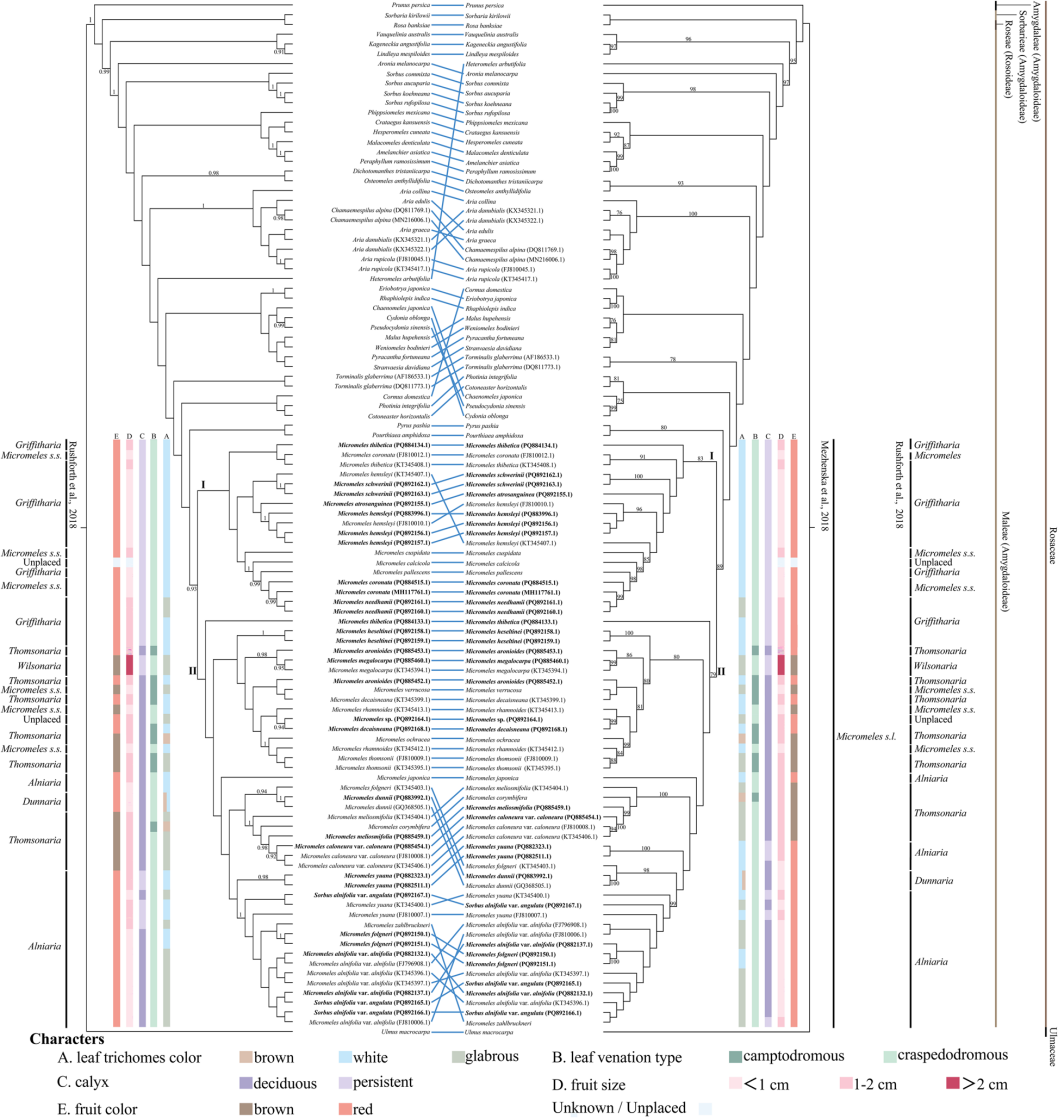
Phylogenetic trees were inferred from the ITS sequcences using Bayesian inference (left) and maximum likelihood (right) methods. Topological differences between the BI and ML analyses are indicated by blue oblique line. Node support values were only labeled for nodes with bootstrap support (BS) ≥ 75% in the maximum likelihood analysis and posterior probability (PP) ≥ 0.90 in the Bayesian analysis. Five morphological characters are mapped on the phylogenetic tree: **A** leaf trichomes color; **B** leaf venation type; **C** calyx deciduous or persistent; **D** fruit size; **E** fruit color

The ITS ML and BI trees exhibited partial incongruence as shown in Fig. 2, necessitating presentation of both topologies. The monophyly of *Micromeles s.l.* is also confirmed in BI (0.93) and ML (89) ITS phylogenies. It is sister to a weakly supported (BI ITS tree, Fig. 2 left) or moderately supported (ML ITS tree, Fig. 2 right) clade including *Pourthiaea* Decne. (*P. amphidoxa* (C.K.Schneid.) Stapf and *Pyrus* (*P. pashia* Buch.-Ham. ex D.Don)). *Micromeles s.l.* is well separated from *Aria* which included only *Chamaemespilus* (100 | 1). While *Torminalis* is inferred as an independent lineage. Two clades, I (0.87/83) and II (0.71/79), are resolved within *Micromeles s.l.* with weak to moderate support. Species relationships within these two clades are more weakly supported than those based on plastome dataset. Furthermore, multiple cases of inconsistent species relationships have been found between the BI and ML ITS trees. Thus, the relationships among the species of *Micromeles s.l*. are not well resolved either in the ITS BI tree or in the ITS ML tree.

### Non-monophyletic species in *Micromeles s.l.*

#### Plastome phylogenies

Nine out of 15 taxa and an identified species sampled by two or more samples in plastome trees are recovered as monophyletic (*M. alnifolia* var. *angulata*, *M. caloneura* var. *caloneura*, *M. coronata*, *M. dunnii*, *M. meliosmifolia*, *M. needhamii*, *M. schwerini*, *M. yuana*, and *Micromeles* sp.), while seven are considered as non-monophyletic (*M. alnifolia*, *M. folgneri*, *M. hemsleyi*, *M. heseltinei*, *M. thibetica*, *M. aronioides*, and *M. decaisneana*).

#### ITS phylogenies

Out of 18 taxa sampled by more than one samples in ITS trees, seven are recovered as monophyletic in both BI and ML trees (*M. caloneura* var. *caloneura*, *M. dunnii*, *M. heseltinei*, *M. megalocarpa*, *M. needhamii*, *M. schwerinii* C.K.Schneid., and *M. thomsonii*). An exception is *M. hemsleyi*, which is non-monophyletic in BI tree, but monophyletic in ML tree.

### Morphological characters

The five morphological characters, leaf trichomes (white, brown, or glabrous) and venation type (craspedodromous or camptodromous), calyx (deciduous or persistent), fruit color (pink, red, or brown), and size (< 1 cm, 1–2 cm, or > 2 cm) are mapped on trees based on both plastome and ITS datasets (Figs. 1 and 2, respectively).

In the plastome phylogeny, all the species in clade I and clade II have leaves with white craspedodromous venation, red fruits (1–2 cm in diameter.) with persistent calyx. However, clade III and clade IV are composed of species with diverse morphologies.

In the ITS phylogeny, all the species in clade I have leaves with craspedodromous venation, red fruits with persistent calyx. However, clade II is composed of species with diverse morphologies.

The AU test of plastome and nuclear data also confirmed the monophyly of *Micromeles s.l.* and the non-monophyletic status of six small genera.

## Discussion

### Phylogenetic inference

Using genome skimming, plastome and ITS datasets were generated in this study. The sampling included representatives of all the seven sections defined by Aldasoro et al. [11], all the six series defined by Yu and Kuan [8], Yu and Lu [9], and six of the seven genera defined by Rushforth [21] in *Micromeles s.l*. The analyses provide well-resolved phylogenetic trees. In congruence with previous studies [24, 28, 46], our results strongly support the monophyly of *Micromeles s.l.* as circumscribed by Mezhenska et al. [3]. Six small genera defined by Rushforth [21] and sampled taxa in this study, *Alniaria*, *Dunnaria*, *Griffitharia*, *Micromeles s.s.*, *Thomsonaria*, and *Wilsonaria*, are non-monophyletic. The sections and series formerly defined by Aldasoro et al. [11] and Yu and Kuan [8], Yu and Lu [9] are non-monophyletic (Figs. 1 and 2). This widespread polyphyly of previously defined series, sections, and subsections based on their morphological characters also appeared in other genera in the same tribe Maleae, such as in *Pyrus* [47] and *Sorbus s.s.* [48].

Incongruence between the plastid and nuclear topologies was observed. This incongruence highlights possible reticulation processes within *Micromeles s.l*., such as hybridization events, chloroplast capture, and introgression, as well as incomplete lineage sorting [26, 49, 50]. Sampling error (i.e., low proportion of informative characters and consequent low resolution levels), especially in ITS phylogeny, may be producing part of the observed incongruence [51, 52]. Similarly, the low proportion of informative characters may be affecting the positions of the different samples representing the same species, as observed in *M. alnifolia* var. *angulata* (Fig. 2). *M. alnifolia* var. *angulata* is represented by three samples collected from CulaiShan and Lushan, Shandong province. It is monophyletic in plastome phylogeny, but is non-monophyletic in ITS phylogeny.

### Genera related to *Micromeles s.l.*

The relationships of *Micromeles s.l.* and its allies of Maleae, however, remain unresolved. Phylogenetic discordances are exhibited in both plastid and ITS trees, and even between different nuclear molecular markers. *Micromeles s.l.* was recovered as sister to the pinnate-leaved *Sorbus s.s.* based on whole plastomes or a few plastid markers [23, 24, 46, 53, 54], but differed in their relationships based on nrDNA data. *Micromeles s.l.* was sister to *Chamaemespilus* based on GBSSI sequence [26], while was sister to either *Aronia* [23] or *Aria* [28] based on ITS sequence. *Micromeles s.l.* and *Sorbus s.s.* form a clade with strongly supported values based on the plastome dataset (Fig. 1), which is also supported in previous studies [48, 53]. The phylogeny based on ITS dataset weakly supported that *Micromeles s.l.* is the sister group of the simple-leaved genera *Pyrus* and *Pourthiaea* (Fig. 2). The conflict between the plastid and nuclear data may be attributed to hybridization, which is prevalent among genera of Maleae [26, 50, 55–58]. The ancestral lineage of *Micromeles s.l.* may have originated from an ancient hybridization between ancestors of the pinnate-leaved *Sorbus s.s.* and some uncertain simple-leaved groups of Maleae [23, 24, 27].

### Species delimitation in *Micromeles s.l.*

The species supported as non-monophyletic in this study call for further taxonomic work in *Micromeles s.l.* This may reflect the difficulties that taxonomists working on *Micromeles* have faced when dealing with species delimitation and identification. Examples concern *M. caloneura*, *M. hemsleyi*, and *M. megalocarpa*. *Micromeles caloneura* is represented by three samples in the plastome phylogeny. One sample of *S. caloneura* collected from Mangshan, Yizhang County, Hunan, is not clustered with the other two samples in clade III and grouped here with three samples of *M. coronata* in clade IV. Morphological re-examination indicates that this specimen (PE 02071916; OR897830.1) matches the diagnosis of *M. caloneura* var. *kwangtungensis*, particularly in its elongated petiole. *Micromeles hemsleyi* is represented by four samples in the plastome phylogeny. One sample (PE 01993957; NC062340.1), stored under the name of *S. hemsleyi* collected from Tianquan County, Ya'an City, Sichuan, is grouped here with two samples of *M. meliosmifolia* collected by the authors from Yingjing County, Ya'an City, Sichuan, in clade III. Morphological re-examination suggests that the specimen, PE 01993957 (cvh.ac.cn), is by the description of *M. meliosmifolia* [59]. The other three samples of *M. hemsleyi* are all grouped in clade IV, but these are non-monophyletic. Both samples of *M. hemsleyi* from Fanjinshan, Guizhou, *X. Chen *et al*., 1594* (OQ100072.1) and *X. Chen *et al*., 1598* (OQ100080.1) are grouped with two samples of *M. needhamii* from Leigongshan, Guizhou, with the one sample of *M. hemsleyi* collected at the type locality of Hubei (OQ100083.1) at the basal lineage. For the three samples of *M. hemsleyi* morphologically agree with the type of this species, further investigation is needed to understand the origin and evolution of this species and its relationship with *M. needhamii*. Two samples of *M. megalocarpa* are isolated in clade III. Further observations reveal that the one collected from Motuo, Xizang (PE 01960095; OR915911.1), is by the description of *M. heseltinei*, although it is not grouped with the other two samples of *M. heseltinei* collected from the same locality. In addition to misidentification, biological factors such as hybridization, incomplete lineage sorting, or polyploidy may also cause the observed non-monophyly.

### Morphological variation

**Fig. 3:**
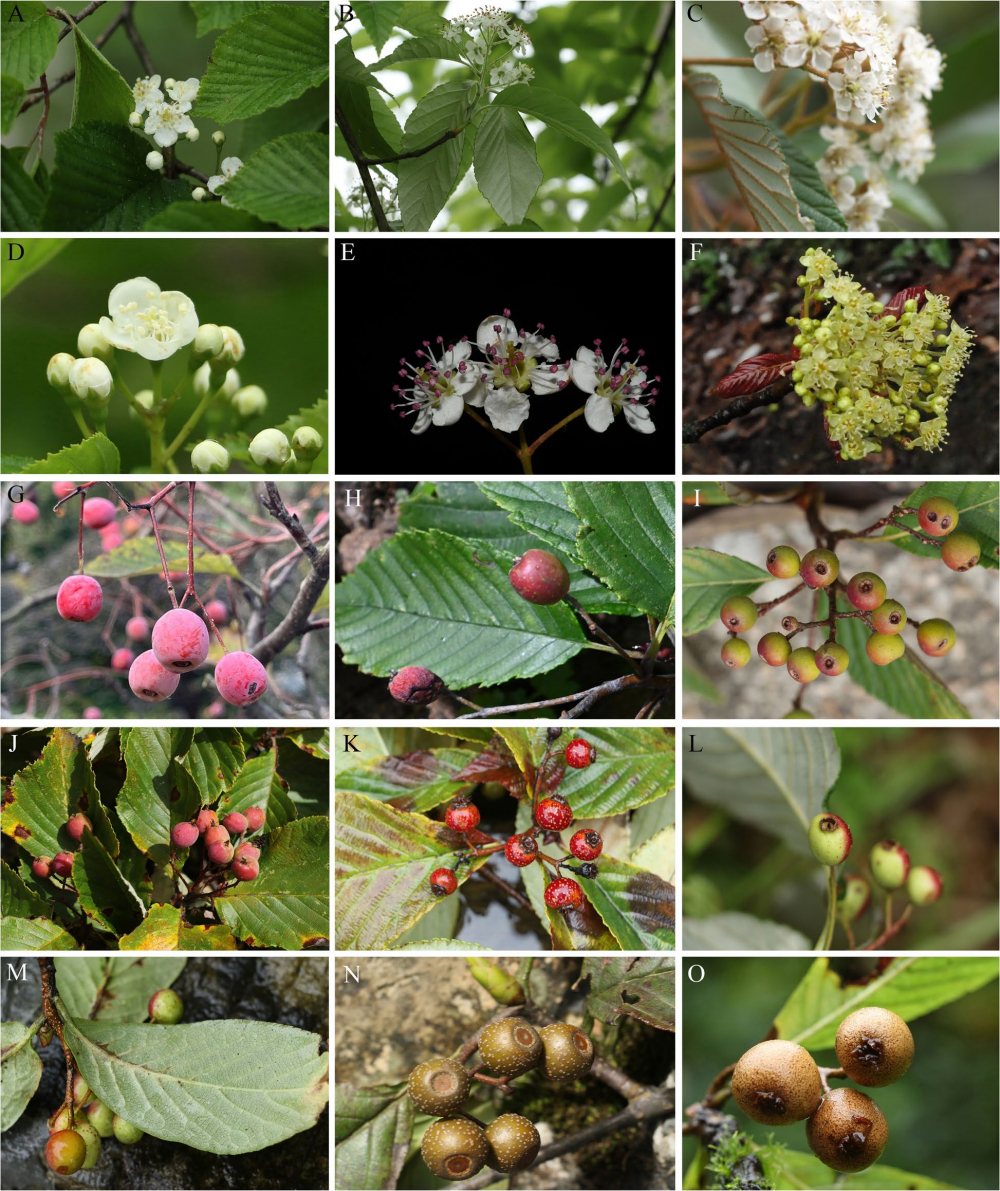
Morphological variation in *Micromeles s.l.* A–F: Variation in leaf trichomes, number of flowers per inflorescence and the color of petals and anthers. A, *M. alnifolia*; B, *M. folgneri*; C, *M. dunnii*; D, *M. alnifolia*; E, *M. caloneura*; F, *M. megalocarpa*. G–O: Variation in leaf venation and fruit morphology. G, *M. alnifolia*; H, *M. yuana*; I, *M. dunnii*; J *M. heseltinei*; K, *M. schwerini*; L, *M. coronata*; M, *M. aronioides*; N, *M. caloneura*; O,* M. megalocarpa*

*Micromeles s.l.* exhibits notable morphological variations in leaf trichomes and venation types, the number of flowers per inflorescence, and flowers and fruit characteristics (Fig. 3).

For example, leaves and inflorescence are subglabrous in *M. alnifolia* (Fig. 3A), those are densely white tomentose in *M. folgneri* (Fig. 3B; Table S2), leaves are densely covered by white tomentose with brown tomentose along veins and inflorescences are covered by densely brown tomentose in *M. dunnii* (Fig. 3C); leaves with camptodromous secondary venation in *M. aronioides* (Fig. 3M), while with craspedodromous secondary venation in other species shown in Fig. 3; the number of flowers per inflorescence range from 6 to 25-flowered in *M. alnifolia* (Fig. 3A) and from 124 to 258-flowered in *M. megalocarpa* (Fig. 3F); petals white, anthers cream in *M. alnifolia* (Fig. 3D), petals white, anthers purplish red in *M. caloneura* (Fig. 3E), petals yellow, anthers pale yellow in *M. megalocarpa* (Fig. 3F); fruit red with deciduous calyx in *M. alnifolia* (Fig. 3G), *M. dunnii* (Fig. 3I), *M. aronioides* (Fig. 3M), red with persistent calyx in *M. yuana* (Fig. 3H), *M. heseltinei* (Fig. 3J), *M. schwerini* (Fig. 3K), *M. coronata* (Fig. 3L), brown with deciduous calyx in *M. caloneura* (Fig. 3N), brown with persistent calyx in *M. megalocarpa* (Fig. 3O); the size of fruit < 1 cm, between 1–2 cm, or larger than 2 cm (Table S2).

Characters, such as leaf trichomes and venation type, calyx traits, fruit color and size, have been considered taxonomically informative [8, 9, 11, 21]. For example, the deciduous calyx has been used to distinguish genera or sections of *Micromeles* by Koehne [12, 13], Yu and Kuan [8], Yu and Lu [9]. However, this is not so with Decaisne, because he included two species with deciduous calyx, *M. alnifolia* and *M. japonica* (Decne.) Koehne, in *Aria* [1]. Phipps et al. [18], Robertson et al. [19] considered that the calyx being deciduous alone is not sufficient to distinguish genera. *Micromeles s.s.* is characterized by the small fruits (4–7 mm in diameter) without lenticels, two co-lateral ovules in the two carpels and generally craspedodromous venation by Rushforth [21]. Yu classified Chinese native simple-leaved species in *Micromeles s.l.* into six series mainly based on venation, indumentum and fruit shape and size [8, 9]. However, Rehder [7] pointed out that the difference in the size of the flowers and fruits is not very marked and has hardly any significance as a generic character. In this study, none of the characters reviewed appears to be unequivocally diagnostic for either of the four major clades inferred from the plastome dataset. Instead, nearly all investigated characters are found in multiple clades across the phylogenetic tree (Fig. 1). As in genera *Pourthiaea* and *Pyrus* in the same tribe Maleae, frequent hybridization-driven morphological transition throughout the evolutionary history may account for this inconsistency in *Micromeles s.l*. [47, 51]. A wide morphological variation presumably facilitates ecological adaptation and may be correlated with the wide species diversification in *Micromeles s.l*.

## Taxonomic treatment

Our results of plastome and ITS phylogenies suggest that the six small genera, *Alniaria*, *Dunnaria*, *Griffitharia*, *Micromeles s.s.*, *Thomsonaria,* and *Wilsonaria*, do not represent natural, evolutionary units, and *Micromeles s.l.* is monophyletic when these six genera are included. Thus, we synonymize *Alniaria*, *Dunnaria*, *Griffitharia*, *Thomsonaria,* and *Wilsonaria* under *Micromeles s.l.* Though the phylogenetic placement of *Pleiosorbus* needs to be tested in future studies, we followed Lu and Ku [60] and Mezhenska et al. [3] to treat it as a synonym of *Micromeles s.l.* for the morphological similarities. An updated taxonomic summary for the expanded genus *Micromeles s.l.* is presented below and new combinations of *Sorbus alnifolia* var. *angulata* and *S. caloneura* var. *kwangtungensis* are proposed.

*Micromeles* Decne., Nouv. Arch. Mus. Hist. Nat. 10: 168. 1874. ≡ *Sorbus* sect. *Micromeles* (Decne.) Rehder, Man. Cult. Trees 382. 1927. ≡ *Sorbus* subg. *Micromeles* (Decne.) Phipps, Robertson & Spongberg in Can. J. Bot. 68: 2244. 1990. – Lectotype (designated by Ohashi & Iketani in J. Jpn. Bot. 68: 357. 1993): *Micromeles rhamnoides* Decne. in Nouv. Arch. Mus. Hist. Nat. 10: 169. 1874 ≡ *Aria rhamnoides* (Decne.) H.Osashi & Iketani in J. Jpn. Bot. 68: 360 (1993) ≡ *Pyrus rhamnoides* (Decne.) Hook.f., Fl. Brit. India 2: 377 (1878) ≡ *Sorbus rhamnoides* (Decne.) Rehder in C.S.Sargent, Pl. Wilson. 2: 278 (1915).

-  = *Alniaria* Rushforth in Phytologia 100: 236. 2018 – Type: *Alniaria alnifolia* (Sieb. & Zucc.) Rushforth in Phytologia 100: 236. 2018
-  = *Dunnari*a Rushforth in Phytologia 100: 240. 2018 – Type: *Dunnaria dunnii* (Rehder) Rushforth
-  = *Griffitharia* Rushforth in Phytologia 100: 233. 2018 – Type: *G. guanii* (Rushforth) Rushforth
-  = *Pleiosorbus* L.H.Zhou & C.Y.Wu in Acta Bot. Yunnan. 22: 383 (2000) – Type: *Pleiosorbus megacarpa* L.H.Zhou & C.Y.Wu
-  = *Thomsonaria* Rushforth in Phytologia 100: 237. 2018 – Type: *Thomsonaria thomsonii* (King ex Hook.f.) Rushforth in Phytologia 100: 237 (2018)
-  = *Wilsonaria* Rushforth in Phytologia 100: 241. 2018 – Type: *Wilsonaria megalocarpa* (Rehder) Rushforth in Phytologia 100: 241 (2018)

Taxonomic notes. – When Decaisne [1] described the genus *Micromeles*, he included five species, *M. verrucosa* Decne., *M. castaneifolia* Decne., *M. rhamnoides*, *M. khasiana* Decne. and *M. griffithii* Decne, but none of them was indicated as the type. When Phipps et al. [18] reduced the genus to a subgenus of *Sorbus*, they designated *Sorbus alnifolia* as type for the name *Micromeles*. The choice of Phipps et al. [18] is to be superseded under Art. 10.2 of *the International Nomenclature Code for algae, fungi, and plants* (*Shenzhen Code*) [61] because it is not one of the original elements of *Micromeles*. Ohashi and Iketani [20] were the first one to achieve to designate the type, *M. rhamnoides*, for the genus name. Later, Aldosoro et al. [11] overlooked the choice of Ohashi & Iketani and chose another original element, *M. griffithii* as the type, and therefore their choice must be superseded.

*Micromeles alnifolia* var. *angulata* (S.B.Liang) Xin Chen, comb. nov. ≡ *Sorbus alnifolia* var. *angulata* S.B.Liang in Bull. Bot. Res., Harbin 10(3): 69 (1990) – Type: China, Shandong, Zhibo shi, Lushan, alt. 600 m, May 1984, *Liang Shu-bin 84,064* (holotype: Herbarium of Shandong Agricultural University!).

Notes. – *Sorbus alnifolia* var. *angulata* was first described by Liang [62]. Liang cited one gathering *Liang Shu-bin 84,064*, deposited at the Herbarium of Forestry school of Shandong province [62]. The type specimen was later transferred to the Herbarium of Shandong Agricultural University when Forestry school of Shandong province was merged with the former. This variety was accepted by Yu and Lu [9] and Lu and Spongberg [45] while Aldasoro et al. [11] treated it as a synonym of *M. japonica*. However, it can be easily distinguished from the latter in having leaves glabrous on both sides at maturity while the leaves hairy adaxially, densely white tomentose abaxially in *M. japonica*. Thus, we accept to treat it as a variety. Accordingly, a new combination, *Micromeles alnifolia* var. *angulata*, is proposed here.

*Micromeles caloneura* var. *kwangtungensis* (T.T.Yu) Xin Chen, comb. nov. ≡ *Sorbus caloneura* var. *kwangtungensis* T.T.Yu in Acta Phytotax. Sin. 8: 223 (1963) ≡ *Thomsonaria caloneura* var. *kwangtungensis* (T.T.Yu) Rushforth in Phytologia 100: 238 (2018) – Type: China, Guangdong (Kwangtung), Lechang (Lok-chang), June 16, 1929, *C.L. Tso 21,123* (holotype: PE-00934273!; isotypes: K-000852618, NAS-00071280!).

Notes. – *Sorbus caloneura* var. *kwangtungensis* was first described by Yu (Yu & Kuan 1963). It was accepted by Yu and Lu [9] and Lu and Spongberg [45] while Aldasoro et al. [11] treated it as a synonym of *M. caloneura*. However, it can be distinguished from *M. caloneura* var. *caloneura* by having much longer petioles and larger red (vs. brown) fruits. Therefore, it is treated as a variety, and a new combination, *Micromeles caloneura* var. *kwangtungensis*, is proposed here.

## Conclusions

The results from plastome and ITS analyses provide new insights into the circumscription of *Micromeles*. *Micromeles s.l.* is a natural group, while the small genera, sections and series previously recognized appear to be non-monophyletic. Most clades and relationships are well-supported in the plastome phylogeny. However, the results show that none of the morphological characters considered taxonomically informative appears to be unequivocally diagnostic for either of the four well-supported clades in the plastome phylogeny. Also, there are phylogenetic analyses revealed for the non-monophyly of seven species. Though the identification of the accessions might need to be further verified, some well-supported incongruence was observed concerning the placement of accessions of the same species between the plastome and ITS trees. Therefore, we propose that deep genome skimming can be useful in further resolving relationships of *Micromeles s.l.* to the remainder of Maleae and relationships between species in the genus. For the accurate delimitation of species boundaries and the understanding of infrageneric classification, we also suggest expanding the taxon sampling to include more accessions collected from type localities and multiple accessions per species collected throughout its distribution range.

## Supplementary Information

Additional file 1: Table S1. Taxon name, voucher, assembly information, and GenBank accession numbers for individuals included in this study.

Additional file 2: Table S2. Information of taxa included in phylogenetic analyses.

Additional file 3: Table S3. Results of alternative phylogenetic hypothesis testing (cpDNA ML).

Additional file 4: Table S4. Results of alternative phylogenetic hypothesis testing (cpDNA BI).

Additional file 5: Table S5. Results of alternative phylogenetic hypothesis testing (ITS ML).

Additional file 6: Table S6. Results of alternative phylogenetic hypothesis testing (ITS BI).

## Data Availability

All 14 newly sequenced plastomes and 30 newly sequenced ITS sequences in this study are available from the National Center for Biotechnology Information (NCBI) (https://www.ncbi.nlm.nih.gov; the accession numbers are distributed across three ranges: PQ899000–PQ899006 (7 accessions), PQ899558–PQ899564 (7 accessions), and PQ882132–PQ892168 (30 accessions); see Table S1: Taxon name, voucher, assembly information, and GenBank accession numbers for the taxa and individuals included in this study). Information for other plastomes and ITS sequences used for the phylogenetic analysis downloaded from NCBI (https://www.ncbi.nlm.nih.gov) can also be found in Table S1. Infrageneric classifications and five morphological features important in distinguishing them in Micromeles s.l. are listed in Table S2.
